# Screening Sonography in the Prehospital Setting: A Case Report on Transforming Clinical Decisions

**DOI:** 10.7759/cureus.91871

**Published:** 2025-09-08

**Authors:** Sergio Miravent, Némis Q Garcia, Inês N Gregório, Bruna S Vaz, Manuel D Lobo, Rui P Almeida

**Affiliations:** 1 Medical Imaging and Radiotherapy, School of Health (ESS-UAlg) University of Algarve, Faro, PRT; 2 Radiology, Basic Emergency Service (SUB) Algarve Local Health Unit (ULS Algarve), Vila Real de Santo António, PRT; 3 Medical Imaging and Radiotherapy, Higher School of Health of the University of Algarve, Faro, PRT; 4 Radiology, Basic Emergency Service in Vila Real Santo António, ULS Algarve, Faro, PRT; 5 Radiology, Northeast Local Health Unit (ULS do Nordeste, EPE), Mogadouro, PRT; 6 Medical Imaging and Radiotherapy, Dr. Lopes Dias School of Health, Polytechnic Institute of Castelo Branco, Castelo Branco, PRT

**Keywords:** ascites, hydronephrosis, hydroureter, screening, ultrasonography

## Abstract

Screening ultrasound has proven highly valuable for the early diagnosis of acute conditions in prehospital settings, particularly in basic emergency services located in peripheral areas with limited access to advanced imaging and laboratory diagnostics. This case report demonstrates the utility of ultrasound in identifying acute medical situations, even in complex patients undergoing oncologic treatment. When performed by radiographers with both theoretical knowledge and hands-on academic training, screening ultrasound can facilitate the early recognition of critical findings and support immediate clinical decisions. The emphasis is not on the long-term management of the underlying pathology but on the decisive role ultrasound plays in guiding acute clinical care, as illustrated by the following case: a patient with cancer, recently operated for an intestinal tumor and receiving treatment for a pelvic neoplasm, presented twice on the same day to a basic emergency service with subtle abdominal pain. On the second visit, a screening ultrasound was central to guiding the patient’s clinical course.

## Introduction

In prehospital emergency services, a brief screening ultrasound performed in the acute setting can rapidly separate patients who require only symptomatic treatment from those who need urgent referral for specialized care [1]. For readers less familiar with the terminology, screening ultrasound refers to a focused near-patient examination designed to answer time-critical questions rather than to replace comprehensive imaging.

Among the wide range of ultrasound findings that may suggest emerging pathology, two are particularly relevant to this case report: free intraperitoneal fluid and hydronephrosis. The simultaneous presence of these findings is uncommon in patients with intestinal (colorectal) carcinoma who also have a neoplastic pelvic mass undergoing treatment [2]; when both are present, ureteral compression by the pelvic mass may occur, together with advanced peritoneal dissemination, requiring a multidisciplinary treatment strategy [3].

Screening ultrasound is particularly well-suited for detecting non-traumatic effusions, such as malignant ascites in oncology patients. The transducer is systematically placed over key anatomical sites, including the hepatorenal recess (Morison’s pouch), perisplenic space, pelvis, and parietocolic gutters. On grayscale imaging, free fluid appears as thin, anechoic layers slipping between mobile bowel loops and solid organs; the hepatorenal angle fills first, and once the volume increases, fluid gravitates into the inferior peritoneal recess [4]. The detection of new-onset ascites in an oncologic patient carries immediate therapeutic implications. Possible underlying causes include peritoneal carcinomatosis, portal hypertension-related decompensation, or chemotherapy-induced hypoalbuminemia. In a low-resource setting, a positive ultrasound scan should timely lead to an early multidisciplinary approach, consideration of diagnostic or therapeutic paracentesis, and timely referral or transfer for further management. A negative study, by contrast, supports continued observation until advanced imaging becomes available. In the same way, a pelvic mass, benign or malignant, may externally compress the distal ureters and bladder, producing upstream urinary stasis.

Hydronephrosis is one of the most readily detected abnormalities on a brief screening ultrasound [5]. The sonogram reveals progressive, anechoic dilatation of the pelvicalyceal system that starts centrally and extends peripherally, creating the characteristic “bear-claw” appearance [6]. Hydronephrosis is typically graded as mild, moderate, or severe based on the extent of calyceal dilatation and the degree of cortical thinning. Since distal ureteral obstruction may be underestimated when the bladder is empty, it is essential for the operator to also assess the bladder. Evaluation with color Doppler to visualize ureteric jets can be informative; absent or significantly asymmetric jets further support the diagnosis of obstruction. Early recognition of hydronephrosis at the bedside prompts timely urological referral, early decompression strategies, and targeted cross-sectional imaging to characterize the underlying pelvic mass [7].

## Case presentation

A 55-year-old female patient presented to a basic emergency service (BES) with right-sided low-back pain for the past four days. Manchester screening (MS) assigns a green (low-urgency) priority under flowchart category 23 for low-back pain, with the discriminator “mild pain < 7 days.” No referral letter accompanies the patient, and vital signs show a tympanic temperature of 36.6 °C and a pain score of 4/10. The patient reported having undergone surgery a month and a half ago for an intestinal adenocarcinoma with a good postoperative outcome. The patient also reported undergoing chemotherapy.

On clinical examination, the abdomen was tender to palpation but showed no signs of peritonism, and bowel transit was normal. The patient received metoclopramide 10 mg intramuscular (IM) and tramadol 100 mg IM. After this medication was administered, the patient’s pain resolved, and she was discharged home in the absence of pain.

Ten hours and 18 minutes after discharge, the patient returned to the BES with a yellow assignment at MS, with worsening of her previous complaints. After the physician’s assessment, an abdominal ultrasound screening was ordered.

**Figure 1:**
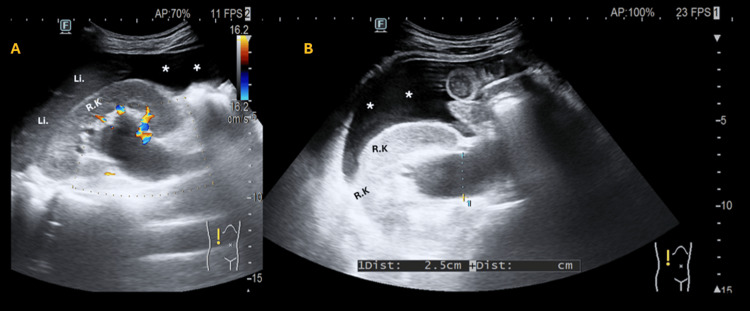
Abdominopelvic ultrasound on the right kidney (hydronephrosis): panels A and B. In panel A, absent Doppler flow confirms true pelvicalyceal dilatation. In panel B, the anteroposterior ureteropelvic diameter measures approximately 2.5 cm, a standard metric used for grading hydronephrosis.

**Figure 2:**
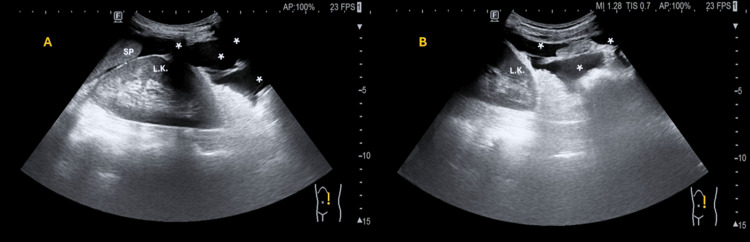
Abdominopelvic ultrasound on a normal left kidney in two longitudinal planes: panels A and B. Sagittal views of the left kidney in panels A and B show a reniform organ with preserved corticomedullary differentiation and an intact echogenic renal sinus  without hydronephrosis. It is important to note that hydronephrosis being only unilateral (on the right) indicates that the mass is not intravesical, that is, the mass is extrinsic to the bladder and slightly to the right sagittal plane. A thin anechoic rim of perinephric free fluid surrounds the lower pole and extends along the lateral margin of the kidney, consistent with free fluid identified by (*). The inferior tip of the spleen (SP) is visualized just superficial to the upper pole of the left kidney and appears sonographically unremarkable.

**Figure 3:**
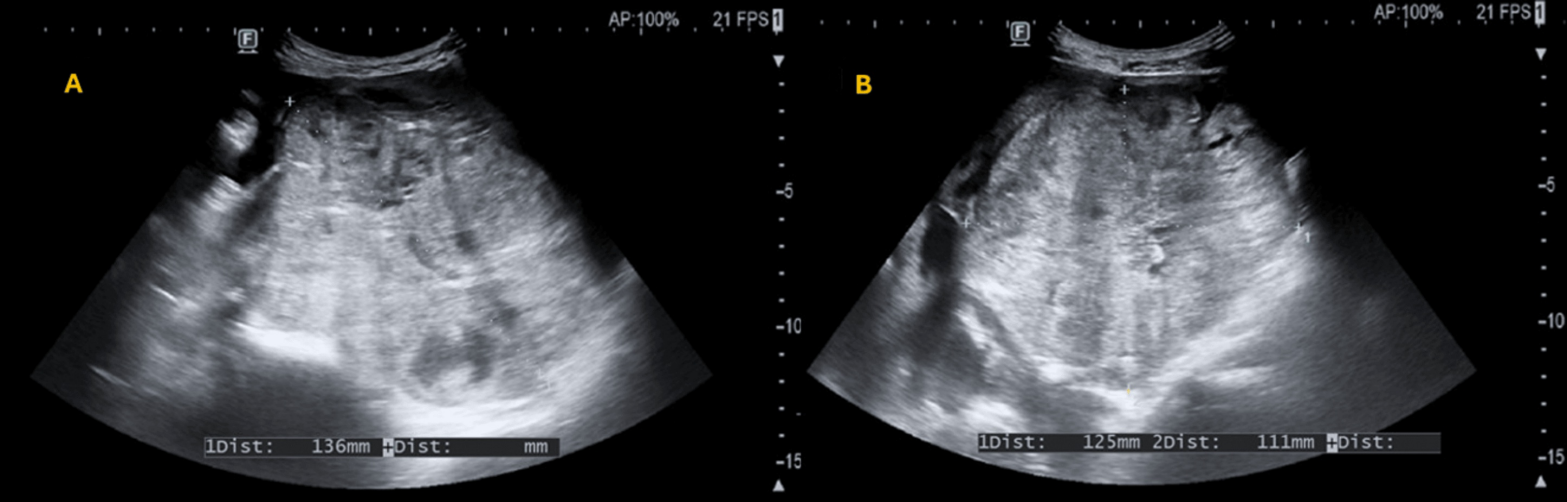
Abdominopelvic ultrasound of biplanar pelvic mass measurements: panels A and B. Panel A (sagittal, long-axis view) shows a well-circumscribed mass occupying the pelvis, measuring 136 mm in the craniocaudal dimension (blue callipers). Panel B (axial, transverse view) shows the same lesion in cross-section, measuring 125 mm in the transverse (left-to-right) axis and 111 mm in the anteroposterior axis. The lesion exhibits mixed echotexture and causes displacement of adjacent pelvic structures, consistent with a space-occupying pelvic mass.

A concise recording of the screening ultrasound is shown through a set of four videos and three still images. Free fluid is present in the superior abdominal recesses as seen in Video 1 (panels A and B) and Video 2 (panel B), respectively. Moderate right hydronephrosis and hydroureter are also seen in Video 2 (panel A). A pelvic mass is visualized in two perpendicular planes in Video 3 (panels A and B). Visual assessment suggests a normal-appearing heart and abdominal aorta in Video 4 (panels A and B). Right-sided hydronephrosis, hydroureter, and pyeloureteric measurements are documented (Figure 1). No sonographic abnormalities were observed in the left kidney (Figure 2). Approximate measurements of the pelvic mass are provided (Figure 3).

**Video 1:** Abdominal ultrasound in the right upper quadrant: panels A and B. Panels A and B display axial recurrent views of the liver (Li) in the right upper quadrant. In panel A, a thin anechoic rim marks free intraperitoneal fluid (indicated by *), the intrahepatic longitudinal portal vein (PV) is visible, and the axial planes of the inferior vena cava (IVC) and extrahepatic aorta (Ao) appear normal. In panel B, the gallbladder (GB), a saccular structure filled with anechoic fluid (bile), appears to float. The patient’s intestinal–pelvic malignancy suggests that the ascites could still be due to peritoneal carcinomatosis.

**Video 2:** Abdominal ultrasound of the right upper quadrant and left upper quadrant: panels A and B. Panel A shows the hepatorenal recess. The liver (Li) is visible only in a small portion superior to the kidney, on the leftmost side of the image, and the right kidney (RK) appears in a longitudinal plane, demonstrating dilation of the renal pelvis and major calyces, while the cortical parenchyma remains preserved, findings consistent with moderate hydronephrosis (grade II). Panel B shows the splenorenal recess, with the spleen (SP) visible. In both panels, (*) indicates free fluid, visualized as an anechoic zone surrounding the respective bilateral organs. A CT scan performed four months earlier showed no free fluid in the superior recess or evidence of hydronephrosis.

**Video 3:** Pelvic ultrasound in two perpendicular planes: panels A and B. Panels A and B show sagittal and axial pelvic sweeps, respectively, where (M) denotes a pelvic mass. Effective chemotherapy rendered much of the tumour necrotic and of low density. Although its overall dimensions increased, the new lower-density composition bulged unevenly, compressing the right ureter and producing the observed hydronephrosis. The well circunscribed margins can be correlate with better subsequent surgery ressection .

**Video 4:** Cardiac subxiphoid ultrasound and aortic ultrasound: panels A and B. Panel A shows a subxiphoid window view, where the liver (Li) serves as an acoustic window. The asterisks (*) indicate a thin anechoic stripe of intraperitoneal free fluid (ascites) located between the liver capsule and the diaphragm. Cardiac anatomy is clearly visualized in a coronal view, including the right atrium (RA), right ventricle (RV), left ventricle (LV) and left atrium (LA). Normal qualitative assessment of cardiac function shows no pericardial effusion. Panel B presents a sagittal view of the abdominal aorta, visualized just  posterior to the liver. A small amount of free abdominal fluid between the liver and diaphragm is also marked with (*).

The attending physician, unable to access digitally the patient’s complete treatment file, retrieved the most recent follow-up computed tomography (CT) study from the Picture Archiving and Communication System (PACS), performed three months after completion of the first chemotherapy cycle and four months before this present BES episode. A CT scan performed four months earlier revealed neither free fluid in the superior recess nor hydronephrosis, as stated in the TC medical report:

"...*The pelvic mass shows an increase in its dimensions, measuring 121 mm × 88 mm in the largest axes in the axial plane (average 87 mm × 66 mm), but it is almost entirely of liquid content, with only a discrete anterior and peripheral uptake area. We assume that this dimensional increase results from the increase in necrotic areas (liquid), with a significant reduction in the solid component, reflecting a response to therapy. There is still an absence of a fatty cleavage plane with the sigmoid loop and with the posterior face of the uterus. No free intraperitoneal fluid is observed. No retrocrural, celiac, mesenteric, lumboaortic, or iliopelvic adenomegaly. Spleen, pancreas, adrenal glands, and kidneys with normal densitometric characteristics*…”

Considering the patient’s condition and the need for a multispecialty comprehensive clinical assessment, the patient was forwarded to the referral hospital. Although the attending physician did not have complete information on her oncologic status, knowing only prior surgery and ongoing chemotherapy for a pelvic mass, the screening ultrasound identified hydronephrosis and new-onset ascites. These acute, actionable findings, together with recurrent pain and malaise, raised concern for obstructive uropathy with potential renal function deterioration and for peritoneal disease or other causes of ascites, thereby mandating urgent reassessment. In view of these risks, definitive imaging and possible decompression were more time-critical than local testing. As laboratory studies obtained at the BES are routinely repeated after transfer and would not have altered disposition, the attending physician prioritised rapid referral over on-site blood work.

## Discussion

Video 1, panels A and B, demonstrates free intraperitoneal fluid. Although no peritoneal implants are visible on initial inspection, the patient’s intestinal-pelvic malignancy makes peritoneal carcinomatosis a plausible cause of the ascites [8,9]. A pelvic mass with right iliac displacement is visible in Video 3, panels A and B, which will later be discussed in the context of unilateral obstructive uropathy, as seen in Video 2, panel A. In Video 2, panel B, the left upper quadrant recess is visualized, with the spleen surrounded by free fluid.

The findings described in the radiologist's report from the CT performed four months earlier are consistent with previously published literature and are consistent with current imaging-based criteria for assessing tumor response to therapy. Even in the presence of an overall increase in lesion dimensions, if such enlargement is attributable to necrotic liquefaction and a concomitant reduction in the viable solid component, it is generally interpreted as a favorable therapeutic response [10,11]. Moreover, this volumetric increase, although secondary to necrosis, may exert a mass effect on adjacent structures, which likely accounts for the right-sided hydronephrosis observed in this patient.

Hydronephrosis can result from oncologic mass effects, with its frequency varying by cancer type and stage, as supported by several studies. In a retrospective analysis, among 44 patients with extramammary Paget’s disease, 13 (30%) developed hydronephrosis [12]. In another study, among 1,409 breast cancer surgeries, seven cases of hydronephrosis associated with recurrence were observed [13]. In bladder cancers, a study of 241 patients undergoing radical cystectomy showed that hydronephrosis was found in 52 patients (21.6%) [14].

Regarding to the patient’s intestinal adenocarcinoma, a retrospective study analyzed 311 patients with locally advanced or metastatic colorectal carcinoma, identifying 39 cases of hydronephrosis diagnosed via CT scan, showing an incidence rate of malignant hydronephrosis of 12.5%, The median age of patients was 43 years, with a range from 23 to 74 years, and the hydronephrosis was unilateral in 29 patients and bilateral in 10 patients. The median time to hydronephrosis development was 17 months [15].

In some cases, the condition may resolve spontaneously or respond to intravenous fluid therapy. In this context, the value of screening ultrasound lies in its ability to distinguish between benign causes of flank pain, such as renal colic or expected discomfort related to oncologic treatment, and urgent, acute conditions like hydronephrosis secondary to an increase in the volume of the mass due to tumor progression, with mass effect (four months before the patient had a tumor and did not have hydronephrosis), which represents tumor progression.

Screening ultrasound should therefore be considered a first-line tool in oncologic patients presenting to urgent care, where seemingly non-specific complaints may mask significant clinical decline requiring prompt detection and intervention.

## Conclusions

Although this acute episode occurred in a cancer patient, which is often more frequent in oncologic and referral hospitals than in BES, it reveals the significant difference in clinical management when a radiographer or sonographer trained in screening ultrasound is available. Compared to on-call physicians without training or access to ultrasound, the presence of a trained operator can substantially enhance clinical assessment. This case highlights how screening ultrasound can fundamentally shift clinical decision-making, leading to more accurate diagnostic suspicions and enabling early identification of acute complications, even when they are secondary to oncologic treatment. However, a key limitation in this case report is the lack of access to the full clinical record from the oncologic referral hospital where the patient was being followed. This absence of longitudinal information may prevent the case from being fully contextualised. Further research is required to determine whether patient outcomes differ between emergency shifts in which a radiographer-sonographer performs screening ultrasound and shifts in which radiographers are present but do not perform screening ultrasound. Beyond the individual case, the findings carry broader implications for emergency system design and patient safety, reinforcing the value of integrating screening ultrasound into routine urgent care pathways.
